# A Case Report of Disseminated Thromboses and Cardiac Ischemia in a Patient With COVID-19 Pneumonia

**DOI:** 10.7759/cureus.39942

**Published:** 2023-06-04

**Authors:** Nehemias A Guevara, Loran Rakovica, Hector Pleitez, Marjorie Mailing Flores Chang, Eduardo Pino-Domenech, Ilmana Fulger

**Affiliations:** 1 Internal Medicine, St. Barnabas Medical Center, Bronx, USA; 2 Internal Medicine, St. Barnabas Hospital Health System, Bronx, USA; 3 Internal Medicine, Texas Tech University Health Sciences Center, Lubbock, USA; 4 Hematology-Oncology, St. Barnabas Hospital Health System, Bronx, USA

**Keywords:** arterial ischemia, thromboses, covid-19 pneumonia, cardiac ischemia, covid 19

## Abstract

The novel coronavirus disease (COVID-19) pandemic caused by the SARS-CoV-2 virus started in December 2019 in the city of Wuhan, in China's Hubei province. This virus quickly spread worldwide, and on March 11, 2020, it was declared a pandemic. Thrombosis, as a hallmark of severe disease, was recognized early as a cause of death; however, the exact pathophysiological mechanism is still not fully understood.

We are reporting the case of a 46-year-old patient who presented with multiple arterial thromboses in the setting of an acute COVID-19 infection requiring systemic thrombolytic therapy and thrombectomy.

## Introduction

The severe acute respiratory syndrome coronavirus 2 (SARS-CoV-2) is a single-stranded RNA coronavirus that enters the human cell mainly through binding to angiotensin-converting enzyme 2 (ACE-2), which is highly expressed in the alveolar cells of the lung, cardiac myocytes, vascular endothelium, and other cells [1].

Since the beginning of the pandemic, multiple hematological disorders have been linked to COVID-19, and thrombosis has been recognized as a hallmark of the disease and one of its deadly complications [2,3]. Furthermore, multiple cases of micro- and macrovascular thrombosis secondary to COVID-19 have been documented, such as pulmonary embolism [4], strokes, venous thromboembolism, cardiac ischemia [5], mesenteric ischemia, and renal artery thrombosis [6-8].

The exact mechanism for thrombosis is still unknown; however, the cytokine storm- the production of interleukin (IL)-2R, IL-6, Il-8, IL-10, and tumor necrosis factor-α (TNF-α) [9]- has been associated with a pro-thrombotic state. Furthermore, the increased activity of the number of the angiotensin (ANG) II/ANG II receptor type 1 (AT1) (due to the loss of function of angiotensin-converting enzyme 2 (ACE2)), in combination with high levels of cytokines, can lead to systemic endothelial injury. It has also been documented that the overexpression of inflammatory mediators in the interstitial space can cause parenchymal damage, a hypercoagulability state, and microvascular and macrovascular thrombosis in the pulmonary and coronary microcirculation [10-12]. Platelet activation and high levels of soluble thrombomodulin, P-selectin, complement 5a, and other inflammatory mediators perpetuate a procoagulant state triggered and maintained by the cytokine storm (with IL-6 playing a central role) [13,14].

We are reporting the case of a 46-year-old patient who presented with multiple arterial thromboses in the setting of an acute COVID-19 infection, requiring prompt intervention with systemic thrombolytic therapy and thrombectomy.

## Case presentation

We present a case of a 46-year-old African male born in Ghana with a previous medical history of unprovoked deep vein thrombosis (DVT) of bilateral lower extremities in 2013, who presented to the emergency department (ED) by ambulance with complaints of right arm cramping and weakness, with a sensation of the arm ‘‘giving up’’ while brushing his teeth. He reported dizziness, blurred vision, and chest pressure with mild shortness of breath over the previous two weeks. Vital signs at arrival were significant for a blood pressure of 216/160 mmHg, a heart rate of 107 beats/min, an oxygen saturation of 82% on room air with a respiratory rate of 18 breaths/min, and a temperature of 97.9 F. Physical examination showed delayed capillary refill on the right (R) radial artery (>2 seconds) and a strong radial pulse on the left (L). Bilateral lower extremity edema was noted. A neurologic examination showed no significant weakness or decrease in sensation.

The chart review revealed that the patient was admitted in 2013 to another hospital for idiopathic bilateral extremity DVT. He was treated with Coumadin and followed up at a Coumadin clinic; a follow-up US Doppler of his lower extremities in March 2014 showed that he had persistent DVT. Coumadin continued for 21 months until May 2015, when the patient lost insurance and stopped the treatment. Thrombophilia work-up in 2013 showed normal protein C and S functional assays and normal cardiolipin IgG and IgM levels, with a negative prothrombin gene 20210 mutation and factor V Leiden. Laboratory abnormalities were significant (Table 1) for an elevated troponin at 1.4 ng/mL, BNP of 240 pg/mL, HCO3 of 20 mEq/L, a mild elevation in creatinine at 1.3 mg/dl, lactic acid at 2.5 mmol/L in venous blood, and COVID-19 PCR was positive.

**Table 1:** Initial basic work-up BNP: brain natriuretic peptide; HCO3: bicarbonate; ALT: alanine transaminase; AST: aspartate aminotransferase

Test	Troponin (0.00-0.49ng/mL)	BNP (0-100 pg/mL)	HCO3 (24-30 mEq/L)	Creatinine (0.6-1.2 mg/dL)	Lactic acid (0.5-2.2mmol/L)	ALT (4-36 IU/L)	AST (8-33 IU/L)	Bilirubin, total (0.1-1.2 mgdl)	White blood cells (4.2-9.1-10*3/uL)	Lymphocytes (21.8-53.1%)
Value	1.4 ng/mL	240 pg/mL	20 mEq/L	1.3 mg/dL	2.5 mmol/L	80 IU/L	65 IU/L	2.5 mg/dl	7.9 x 10*3/uL	1.01%

**Figure 1:**
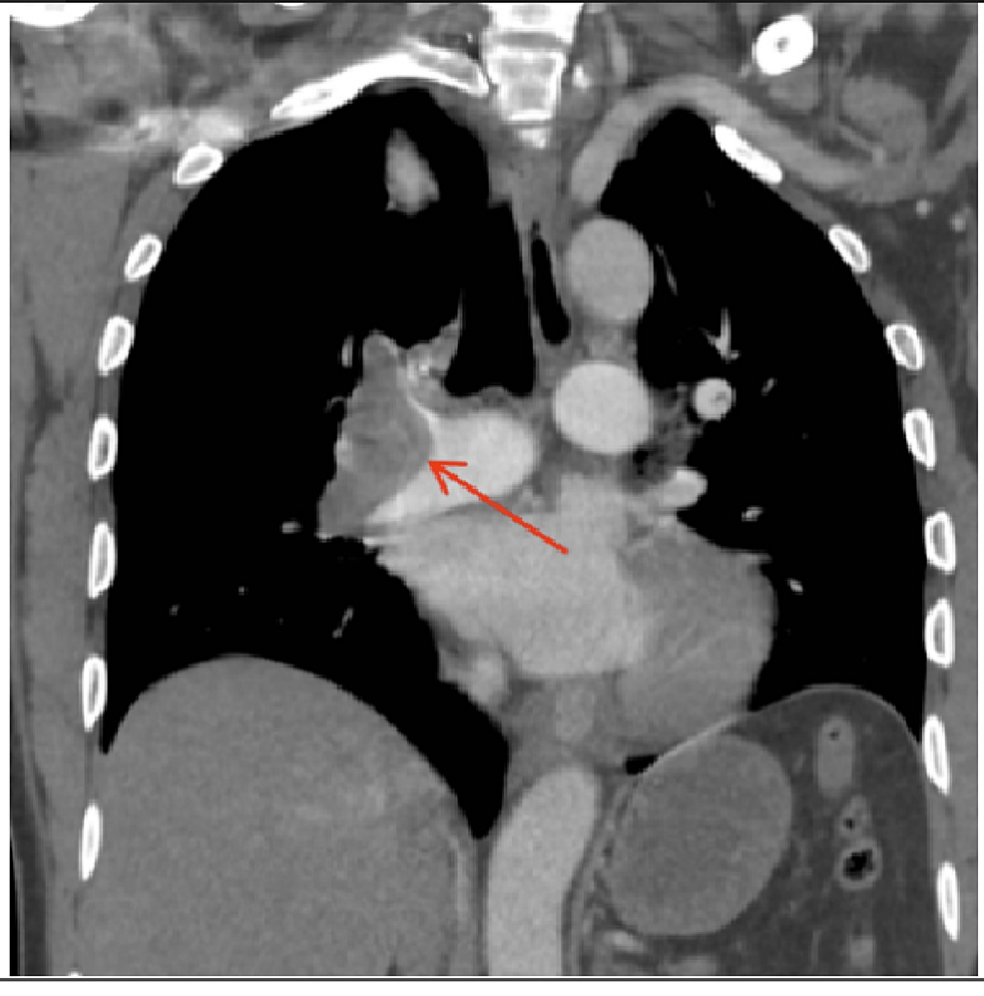
Coronal CT showing right (R) pulmonary artery embolism

The initial work-up showed an electrocardiogram (EKG) with ventricular bigeminy and a 96 beats/min heart rate. The chest X-ray at the initial work-up was significant for prominent central hila. The calculated Wells score for pulmonary embolism (PE) was six points. Therefore, urgent computer tomography (CT) with contrast of the upper extremities, CT angiography (CTA) of the chest, and bilateral ultrasound (US) Doppler of the upper and lower extremities were performed. A heparin drip was initiated because of the high probability of arterial thrombosis. The patient was found to have a pulmonary embolism (Figure 1).

**Figure 2:**
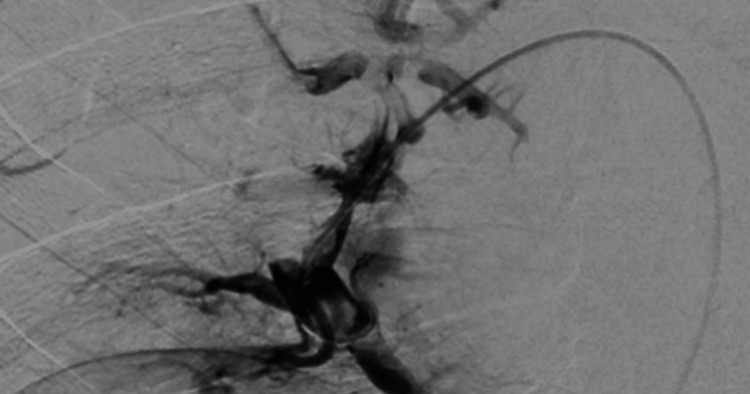
Pulmonary angiogram showing pig-tail catheter thrombolysis; note the smaller pulmonary embolism, likely due to mechanical disruption prior to catheter-guided thrombolysis

Furthermore, CT with contrast of the upper extremities demonstrated the presence of contrast in venous structures and no definite vascular abnormalities. CTA of the chest (Figure 2) showed multiple pulmonary emboli, with a significant filling defect in the distal right main pulmonary artery and extension of emboli into the pulmonary arterial branches in the right upper lobe, lower lobe, and right middle lobe. Therefore, an interventional radiology service was consulted, and a pulmonary angiogram with mechanical disruption of the pulmonary embolism was performed. Thrombolysis with alteplase infusion via a lysis catheter was placed in the R common femoral vein with resolution of the thrombosis (Figure 2).

Troponin levels peaked at 80 ng/mL and then decreased to 10.64 ng/mL within four days (Table 2). Cardiology was consulted, and demand ischemia was suspected.

**Table 2:** Troponin trend

Date	01/19/2022	01/20/2022	01/20/2022	01/21/2022	01/23/2022
Troponin (0.00-0.49ng/mL)	1.43 ng/mL	>80.00 ng/mL	>80.00 ng/mL	24.24 ng/mL	10.64 ng/mL

**Figure 3:**
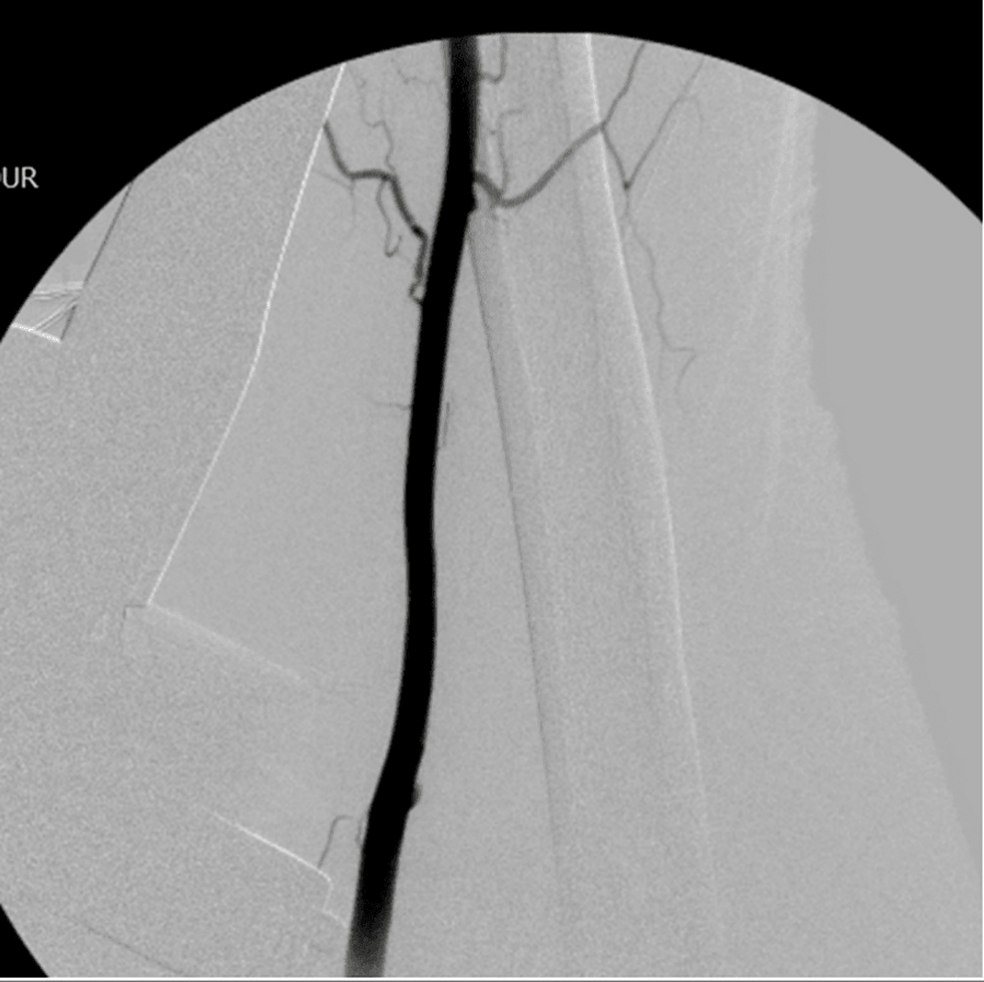
Post-surgery angiogram showing resolution of occlusion on the right brachial artery

A follow-up physical examination eight hours after the directed catheter thrombolysis was remarkable for gross deficits in the right upper extremity with a strength of 3/5 and an absent radial pulse with intact sensation. Therefore, arterial US Doppler of the upper extremities was performed, showing occlusion of the right axillary artery and proximal portion of the right brachial artery, with a diminished flow in the remainder of the right brachial, radial, and ulnar arteries, likely from the more proximal occlusion in these arteries. Vascular surgery was consulted urgently for R-arm arterial occlusion. An embolectomy with a Fogarty catheter was performed immediately. A large clot was removed from the right brachial artery and sent for pathology examination. This clot was found to measure 5x1x1 cm and weigh 2.5 grams. Palpation of the clot showed no solid tissue, and no microscopic examination was done. A post-surgery angiogram showed a good, brisk flow of the R-arm (Figure 3). The patient was started on a heparin drip and modified as per protocol.

Because of the upper extremity’s thrombosis, an interatrial shunt was suspected. Therefore, transthoracic echocardiography with the bubble technique was performed and showed an interatrial shunt. Repeated thrombophilia workups were negative (Table 3).

**Table 3:** Secondary thrombophilia workup

Antithrombin Ag	Protein S-function	Protein C-function	Factor II DNA	Factor V Leiden
107%	63%	110%	Not detected	Non-detected

Moderate transaminitis was evident from day one of hospitalization (Table 1), with a peak on day two (alanine transaminase (ALT) of 160 IU/L and aspartate aminotransferase (AST) of 438 IU/L). Therefore, Budd-Chiari syndrome was considered and ruled out with the US Doppler abdomen in the context of multiple thromboses. Secondary causes of elevated liver function tests were ruled out (Table 4), and the transient transaminitis was suspected to be associated with the ongoing COVID infection.

**Table 4:** Secondary causes of transaminitis evaluation

Hepatitis A/B/C/E Ag/Ab (negative-positive)	Alpha-1-antitrypsin (101-187 mg/dl)	T-transglutaminase (0-5 U/mL)	Ceruloplasmin (16.0-31.0 mg/dL)	Mitochondrial (m2) antibody (0-20 units)	Actin (smooth muscle) antibody (0-19 units)	Liver -Kidney microsomal antibody (0.00-20.00 units)
Negative	119 mg/dL	<2 U/mL	26.5 mg/dL	<20 Units	6 Units	<20.1 Units

The patient was discharged with a resolution of the acute symptoms and normalization of creatinine and liver function tests. Furthermore, oral anticoagulation was started on day four of hospitalization with apixaban (10 mg twice daily). The patient currently follows up with the hematology clinic at our institution.

## Discussion

Since the beginning of the COVID-19 pandemic, thrombotic events have been noted in up to one-third of cases [15]. Venous thromboembolism (VTE) has been the main thrombotic event related to COVID-19 [16]. The coagulopathy of COVID-19 infection is complex, involving an interplay between endothelial cell injury, inflammation, and coagulation. VTE rates are higher in severe COVID-19 cases, suggesting that the high incidence is due to the combination of VTE risk factors in hospitalized patients and COVID-19-induced coagulopathy [3].

The SARS-CoV-2 infection requires the co-expression of ACE2 receptors and a transmembrane protease serine 2 (TMPRSS2). The ACE2 receptor is expressed in endothelial cells, cardiomyocytes, pulmonary epithelial cells, pulmonary vasculature, kidneys, adipose tissue, and the central nervous system, which may contribute to extrapulmonary thrombotic complications [12,17]. Two mechanisms can cause endothelial damage due to SARS-CoV-2 infection: direct invasion of endothelial cells or indirect hyperinflammation mediated by IL-1, TNF-a, and IL-6. These mechanisms lead to activation of the extrinsic or tissue factor (TF) pathway, exocytosis of Weibel-Palade bodies (WPB) from endothelial cells, enhancement of the expression of P-selectin and E-selectin, and recruitment of neutrophils, monocytes, and macrophages [12]. This phenomenon is known as thrombo-inflammation or COVID-19-associated coagulopathy (CAC) [18].

In addition, neutrophil extracellular traps (NETs) are a form of decondensed chromatin extruded by dead or dying neutrophils, which capture SARS-CoV-2 and promote thrombus formation by activating the intrinsic pathway and platelets. Activated platelets promote pathogen clearance by forming platelet aggregates and microthrombi [12]. The contribution of complement-mediated endothelial injury has been suggested. An in vitro study found that SARS-CoV-2 spike protein could activate the complement component (C5a) and membrane attack component (C5b-9) in severe COVID-19-infected patients, suggesting "endotheliopathy" as the primary contributor to COVID-19-related severity and mortality [19,20].

Myocardial injury and arrhythmias have been demonstrated in patients with COVID-19. The mechanisms of elevated troponin in COVID-19 are likely to be multifactorial. The increased inflammatory and thrombotic responses following COVID-19 infection increase the risk for acute nonischemic myocardial injury and acute myocardial infarction, particularly Type II. In general, for any patient with increased troponin above the 99th percentile, myocardial injury can be classified as chronic, acute nonischemic, or acute myocardial infarction (MI) [21-23].

A study that analyzed electrocardiographic findings demonstrated that only 4% of patients presented with premature ventricular contractions (PVC) [21]. Atrial fibrillation, flutter, left bundle branch block, and electrocardiogram signs suggesting acute right ventricular pressure overload, PVC, and ST-segment deviation have all been associated with elevated troponin levels and mortality in COVID-19 patients. Critical illnesses such as acute pulmonary embolism and sepsis can also cause troponin increases without overt myocardial ischemia, categorized as acute nonischemic myocardial injury [12,22].

In a prospective cohort of patients with COVID-19, the cumulative 90-day incidence of venous thromboembolism ranged from 0.2% to 0.8% among all cases and 4.5% for those hospitalized. Arterial thromboembolism ranged from 0.1% to 0.8% among all cases, increasing to 3.1% among those hospitalized. The occurrence of venous thromboembolism in patients with COVID-19 was associated with an increased risk of death for patients not hospitalized or hospitalized, as was the occurrence of arterial thromboembolism, respectively (3.16 (2.65-3.75) and 1.93 (1.57-2.37)) [3].

A large retrospective study looking for the association of COVID-19 with major arterial and venous thrombotic diseases showed a higher risk for first arterial thrombosis (three to six folds) compared with venous thrombosis (two to three folds) within the first week after COVID-19 diagnosis and a decline after the first week of diagnosis. However, the risk of arterial thrombosis can increase up to 49 weeks after COVID-19 diagnosis. In addition, after the first week of diagnosis, the risk of venous thrombosis is higher than that of arterial thrombosis [24-26]. The risk of arterial thrombosis is higher in the extremities compared to the core organs (heart, kidney, brain, etc.). However, the lower extremities are more frequently affected (71%) compared with the upper extremities (14%). It is important to note that patients with arterial thrombotic events will show minimal or no respiratory symptoms [24,27].

The majority of outpatients with COVID-19 do not require anticoagulation. However, outpatient thromboprophylaxis may be appropriate for selected individuals with COVID-19, especially those with prior VTE or recent surgery, trauma, or immobilization. The ACTIV-4B Outpatient Thrombosis Prevention Trial supports not using anticoagulation or antiplatelet agents in outpatients without established risk factors [28]. In addition, the practice of not using anticoagulation in most outpatients is consistent with several expert panels [29].

The American Society of Hematology (ASH), in its last update of May 2022, suggests prophylactic intensity over therapeutic intensity anticoagulation at most for patients with COVID-19-related critical illness who do not have suspected or confirmed VTE. However, this is a conditional recommendation based on very low certainty in the evidence about the effects [29].

The most common causes of upper extremity thrombosis are traumatic upper extremity injuries or embolisms. The main indication for upper extremity revascularization is acute limb ischemia. However, determining whether an intervention is necessary is based on the clinical characteristics of every patient [30]. In most patients with COVID-19 and acute, objectively confirmed pulmonary embolism not associated with hypotension, the CHEST Guidelines and Expert Panel recommend against systemic thrombolytic therapy and anticoagulation therapy for a minimum duration of three months [31].

This is an atypical presentation of a 46-year-old male with acute COVID-19 infection with pneumonia, pulmonary embolism, and disseminated arterial and venous thrombosis without identifiable risk factors such as inherited thrombophilia, obesity, smoking, diabetes, immobility [12,17], and myocardial injury. Furthermore, the work-up showed a positive echocardiogram bubble study, which explained his presentation with a right arm arterial thrombosis due to a paradoxical right-to-left embolization via an interatrial defect. Our patient is currently following up as an outpatient with hematology-oncology and taking daily, lifelong prophylactic apixaban without complications.

## Conclusions

Coagulopathy manifestations are a hallmark of the COVID-19 disease; however, multiple thromboses, such as deep venous thrombosis, pulmonary embolism, and arterial thrombosis, are not usually seen. This case highlights the importance of an interdisciplinary approach to patients with a high burden of thrombosis due to COVID; prompt treatment is imperative to avoid deadly complications.
